# Study on the infrared dynamic evolution characteristics of different joint inclination phyllite under uniaxial compression

**DOI:** 10.1038/s41598-023-37098-w

**Published:** 2023-06-18

**Authors:** Xie Chengyu, Lan Weihang, Chen Ziwei, Wu Yabin

**Affiliations:** 1 School of Environment and Resources, Xiangtan University, Xiangtan, 411105 China; 2 Changsha Institute of Mining Research Co., Ltd., Changsha, 410012 China

**Keywords:** Solid Earth sciences, Engineering

## Abstract

The destructive behavior of rocks and the evolution behavior of cracks are highly correlated. With the continuous development process of crack, the stress state of rock is constantly broken until entirely failed, so it is necessary to study the spatial and temporal behavior characteristics of the crack in the process of rock destruction. In this paper, the destruction process of phyllite specimens is analyzed by thermal imaging technology, and the temperature evolution process of the crack is studied to explore the infrared characteristics of the crack evolution process. Furthermore, a model for predicting rock destruction time is proposed based on Bi-LSTM recurrent neural network model combined with Attention mechanism. The results show that: (1) During the development of rock cracks, the rock surface shows a stable dynamic infrared response, and shows different evolutionary characteristics in different stages, mainly including temperature reduction in the compaction stage, temperature rise in the elastic and plastic stages, and temperature peaks in the failure stage; (2) During the evolution of the crack, rock destruction has a significant control effect on the IRT field along the fracture tangential and normal direction, and its distribution has the volatility controlled by the time; (3) The recurrent neural network method is used to predict the rock failure time, the results can be used as a method to predict the time of rock destruction, and it can be further put forward the corresponding protective measures accordingly, to maintain the long-term stability of the rock mass.

## Introduction

As a gradual mechanical behavior, rock destruction is essentially a process to generate, expand, and coalesce microcracks inside the rock^1^. The long-term stability of the rock is of great significance to slope stability, roadway support, and geotechnical engineering construction. It is the main direction of the current research by clarifying the destruction law of different rocks and proposing the corresponding protection methods.

At present, the main crack detection methods are the acoustic emission (AE), microseismic (MS) monitoring, and digital image correlation (DIC) techniques. AE/MS identifies and detects the defects and cracks of the rock mass by capturing the elastic waves generated by the acoustic transmitter and the rock mass fracturing themselves^2–6^, while DIC obtains the rock mass's deformation information by comparing the digital images before and after the deformation^7,8^.

Infrared thermal imaging technology, by accepting the infrared radiation energy emitted from the surface of the observed object, the size of the energy value is related to the temperature and radiation rate, which provides a new perspective for the detection method of rock pores, cracks^9–17^. This method can realize the non-destructive and^18^ non-contacting detection of rocks, which can be combined with AE and other techniques^17,19^, or define quantitative indicators to analyze the rock destruction process. Previous scholars have carried out research on sandstone^9,10,20,21^, coal, granite^22,23^. In the quantitative treatment of thermal images, Wu et al.^10^ used AIRT to explain the destruction process of rocks and proposed three infrared precursors in the rock evolution and Zhang et al.^21^ proposed that CIRT and SIRT studied the defective sandstone. By analyzing the time curve of the quantitative index, the abnormal changes of the IRT field before rock fragmentation are analyzed, and then the prediction model and the fitting equation are proposed.

Simulation prediction of rock destruction using an engineering model and machine learning are effective methods to predict the failure process of rock^24–27^. Due to the influence of the environment, the rock failure curve has a strong nonlinear relationship. The deep learning method can provide better flexibility and accuracy with its excellent fault tolerance, learning, and generalization ability^28^. By calculating the limited information, the compressive strength of rock^11,29,30^, stress–strain curve^31,32^, and break time^21^ is predicted in advance.

Previous studies mainly studied the infrared development characteristics of rock whole^10,21,33^, but for different rocks, due to their composition, arrangement, internal gap, and destructive behavior being different, the surface emission rate is not consistent, resulting in the temperature trend varies significantly between different rock species and different regions, and the rock with a weak structural surface is less studied in previous studies. Therefore, we use thermal imaging technology to study the infrared evolution characteristics of rock with different joint dip angles. By defining the infrared radiation characteristics, the infrared changes of different failure processes of rock are explained and the differences and connections between the infrared evolution of jointed rock and other rock masses are discussed. Finally, by combining a variety of infrared analysis indicators defined by us, we use the Bi-LSTM neural cycle network combined with Attention mechanism to predict the failure time of rock, and compare it with other models to verify the advantages of our model, which expands the application of neural network technology in thermal imaging technology.

## Materials and methods

### Experimental design

#### Experimental equipment

**Figure 1:**
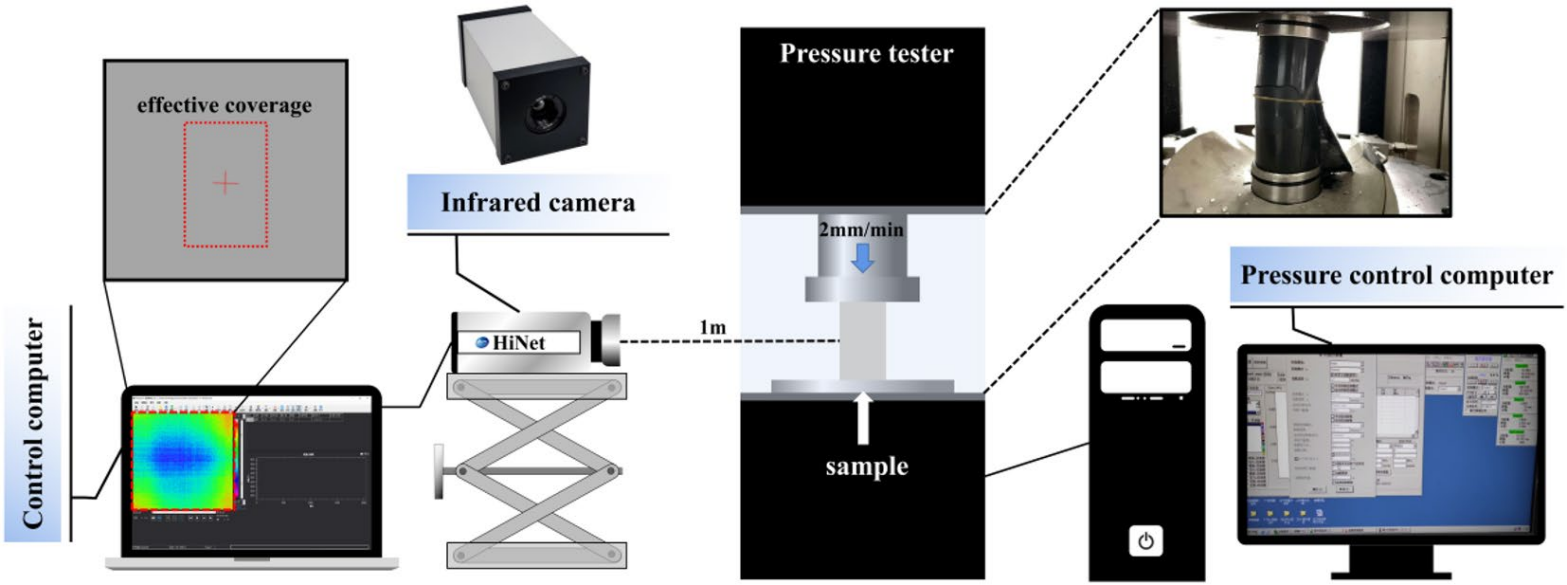
Schematic diagram of the experimental arrangement.

In order to capture the infrared characteristics of the process of rock damage, the objective connection between stress and infrared radiation is explored. We perform uniaxial compression of phyllite (cylinder) specimens with different inclination angles in moist state, and the specific scheme is as follows (Fig. 1):

1. The MTS815 rock mechanics test system (manufactured by MTS Systems Corporation) has the maximum axial pressure of 4600 kN, and the measurement accuracy is within ± 0.5%. The displacement control is set at 0.2 mm/min to compress the sample.
2. HiNet384/640 online measurement of infrared thermal imager, using low noise non-refrigeration infrared core, can generate a thermal image of 640 × 512 pixels, the temperature measurement range is (− 20 to  + 550) °C, the pixel spacing is 17 μm, the response band is 8–14 μm, and the error is less than ± 2%. In the experiment, the additional frame rate was set at 10 Hz, the focal length was 1 m, the temperature gain was set to the high-gain mode, the thermal emissivity and transmission rate were set to 1.00, and the tropical strips were controlled at the ambient temperature of ± 2 °C.
3. The size of the phyllite specimens used is 50Φ × 100 (mm), the pedion error does not exceed ± 0.1 mm, the perpendicularity is within 0.25%, and the inclination error is less than 0.5°, which is in line with the recommended size and error range proposed by the International Society for Rock Mechanics. In order to ensure uniform axial force and reduce the influence of anisotropy on the experimental results, we polished the surface of the specimens. It was also placed in the laboratory for 24 h before the experiment to reduce the impact of environmental factors. Sample parameters are shown in Table 1.

**Table 1:** Geometries of phyllite specimens.

Number	Dip angle (°)	Diameter (mm)	Altitude (mm)	Wave speed (km/s)	Density (kg/m^3^)	Porosity (%)
Sample 1	30	49.97	100.02	3.03	2.423	6.81
Sample 2	45	49.99	100.07	4.55	2.423	6.81
Sample 3	60	50.02	99.97	5.40	2.423	6.81
Sample 4	0	50.10	100.10	2.88	2.423	6.81
Sample 5	90	49.92	99.98	4.88	2.423	6.81

#### Experimental arrangement

Place the specimen on the lower platen, and the center of the specimen shall be aligned with the center of the lower platen. The position and height of the thermal imager and the camera device are arranged so that the lens and the specimen are at the same height in the vertical direction, and the horizontal direction distance is 1 m. To increase the contrast between the rock sample and the environment and to facilitate focus, the rock mass surface was slightly moistened with water before the start of the experiment. Meanwhile, due to environmental disturbances, the thermal imager will automatically focus, causing data mutations.

Considering the change of the thermal radiation has strong sensitivity, the experiment time is set in the morning when the ambient temperature is low. On the night before the experiment, the experiment strictly forbade personnel access, closed the doors, controlled the wind. During the experiment, people are forbidden to walk around, and the test piece can not be removed slowly until the end of the experiment process.

### Replicate the spatial distribution of IRT fields using Python

**Figure 2:**
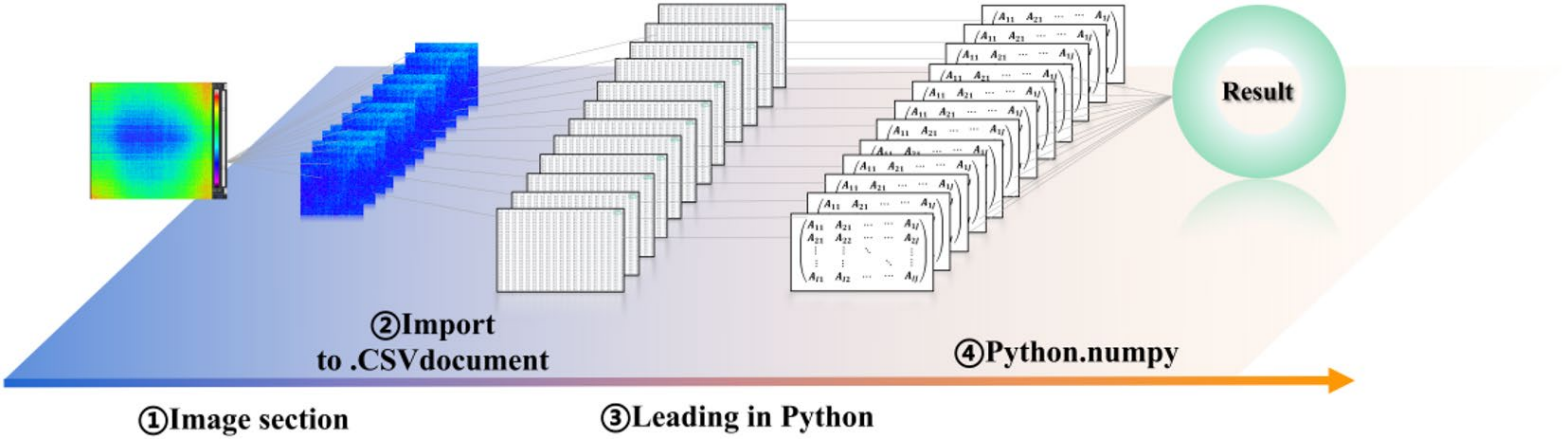
Flow chart of IRT field quantitative indicators.

The HiNet384/640 thermal imager can collect the object surface temperature information at any time and save the collected information in a file in RAW format. Each thermal image consists of a spatial matrix of 640 × 512, and each element of the matrix represents the temperature value of the corresponding pixels of the image. During the experiment, the disturbance of the environment and the changing temperature of the rock surface leading to the temperature distribution recorded at each time is different. Comparative analysis of image evolution in time shows the behavioral characteristics of thermal accumulation during rock destruction. This paper proposes a method to implement this operation using Python, specifically (Fig. 2):

1. The thermal image sequence is sliced by the thermal image instrument control system to obtain the experiment’s required fragment sequence and save it into a series of files in CSV format.
2. Sequence files are filtered out and read in using the standard library (-OS) of Python.
3. Each spatial matrix is traversed by Python's extended library, and the data is processed by a built-in function to find the maximum, minimum, mean, and standard deviation of the matrix and stored it in a list.
4. The resulting sequence results are finally visualized.

### Establishment of the IRT field quantitative indicators

Based on statistics, previous scholars have proposed a series of quantitative analysis indicators^11,20,21,33^. By comparing each element (T_ij_, i is the number of rows in the matrix and j is the number of columns of the element in the matrix) on the spatial matrix (A(I × J)), five main indexes, namely MAXIRT (the maximum value), MINIRT (the minimum value), AIRT (the mean value), CIRT (standard deviation) and SIRT (asymmetry), which are defined as follows:

1 $$ MAXIRT = {\text{max}}\left( {A\left( {I \times J} \right)} \right) $$

2 $$ MINIRT = {\text{min}}\left( {A\left( {I \times J} \right)} \right) $$

3 $$ AIRT = \frac{1}{I \times J}\sum\nolimits_{i = 1}^{I} {\sum\nolimits_{j = 1}^{j} {A_{ij} } } $$

4 $$ CIRT = \frac{1}{AIRT}\sqrt {\frac{1}{I \times J}\mathop \sum \limits_{i = 1}^{I} \mathop \sum \limits_{j = 1}^{J} \left( {A_{ij} - AIRT} \right)^{2} } $$

5 $$ SIRT = \frac{1}{{AIRT^{3} }}\frac{1}{I \times J}\mathop \sum \limits_{i = 1}^{I} \mathop \sum \limits_{j = 1}^{J} \left( {A_{ij} - AIRT} \right)^{3} $$

According to the results analyzed by the scholars^21^, this paper finds that some quantitative indicators (MAXIRT, MINIRT, SIRT) are not suitable for the analysis of rocks with some nodal dip of joint in terms of their time evolution. Meanwhile, AIRT has temporal visibility and stage change characteristics^10,34^, and can also reflect the load and axial stress state, so AIRT and CIRT is used as the quantitative description of the distribution characteristics of rock in time.

## Results

### Failure characteristics of different section angles

**Figure 3:**
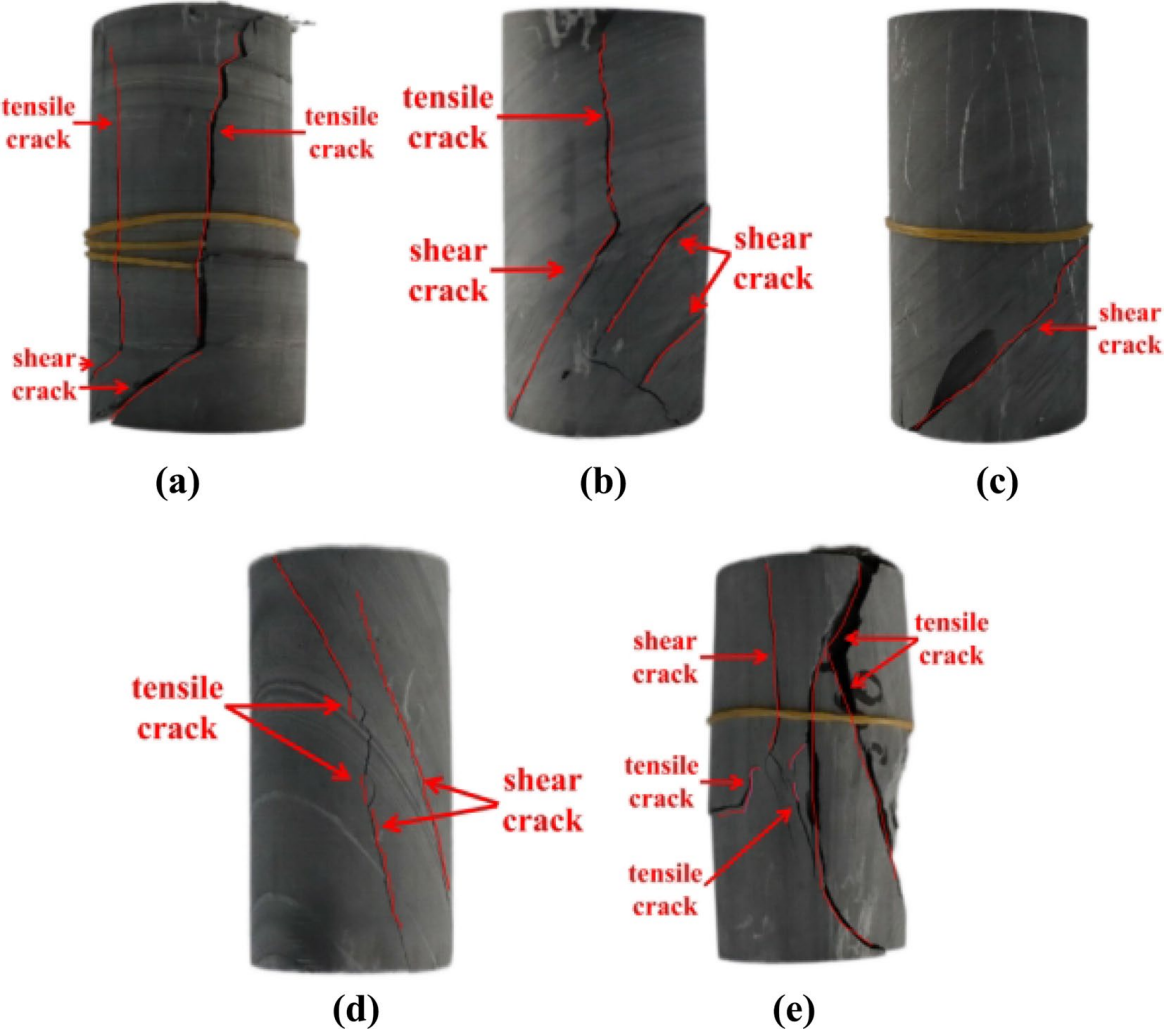
Phyllite failure mode under uniaxial compression: (**a**–**e**) represent damaged phyllite specimens of 0°,30°,45°,60°,90°, respectively.

Figure 3 corresponds to the failure mode of phyllite at different dip angles under uniaxial compression. The failure cracks of the phyllite specimen with 0° are tensile and shear cracks, and the final failure mode after compression is mainly tensile-shear compound failure mode; for samples with 45° and 60°, the main crack form is a shear crack, and the crack direction is mainly parallel to the weak surface; in samples with a joint inclination of 90°, the final failure mode is mainly a tensile-shear compound failure. Therefore, with the increase of the angle of phyllite, the angle control ability of the crack direction changes from weak to strong to weak and reaches the maximum value at 45°.

The number of cracks shows a monotonous increasing trend for the phyllite specimens with different inclination angles as the pressure was continuously accumulated. From the degree of crack extension, the samples with joint inclination of 45° and 60° have shear cracks in the direction along the structural weak plane but produce no obvious cracks in the rest of the site. For the sample with a joint inclination of 0° and 90°, there are more micro-cracks, the crack expansion degree is more thorough, and the structural failure is fuller after the damage. At the same time, due to the contact friction of the upper platen, the top appeared a penetrating tensile crack. Obviously, at 45°, the change of the joint inclination will promote the extension of the crack and reach the peak at 0°/90°.

Table 2 records the uniaxial compression results of different samples. Sample with a joint inclination of 90° has the highest compressive strength, followed by sample with a joint inclination of 0°. The sample compressive strength is the lowest at the joint inclination of 30°. When the joint inclination gradually increases from 30° to 90°, the compressive strength also continuously increases. The main reason for this phenomenon is that when the rock inclination angle is 0°/90°, the state of compression direction and joint angle is vertical and parallel, respectively, and limited by the pressure plate, the axial displacement is limited between different layers. When the inclination angle is 0°–90°, the difficulty of shear sliding between the surface of the layers is different, and reach the minimum value when the inclination angle is 30°–45°. According to the single discontinuity theory, When the weak plane of the structure is perpendicular to the direction of the maximum principal stress, the compressive strength of the rock mass is the strength of the rock itself, and when the rock is staggered along the weak plane, the compressive strength of the rock mass is the compressive strength of the weak plane. When the rock inclination angle and the compression direction are at a certain angle, the control ability of the weak surface of the structure on the compressive strength of the rock is stronger. Therefore, as the inclination angle increases, the compressive strength of the sample shows a U-shaped trend, which is similar to the previous research results^35^.

**Table 2:** Uniaxial compression results of phyllite with different inclination angles.

The specimen number	Load time (s^−1^)	Compressive strength (MPa)
Sample 1	76	18.66
Sample 2	92	38.98
Sample 3	83	39.11
Sample 4	193	79.60
Sample 5	193	111.41

### The destruction process of different section angles

**Figure 4:**
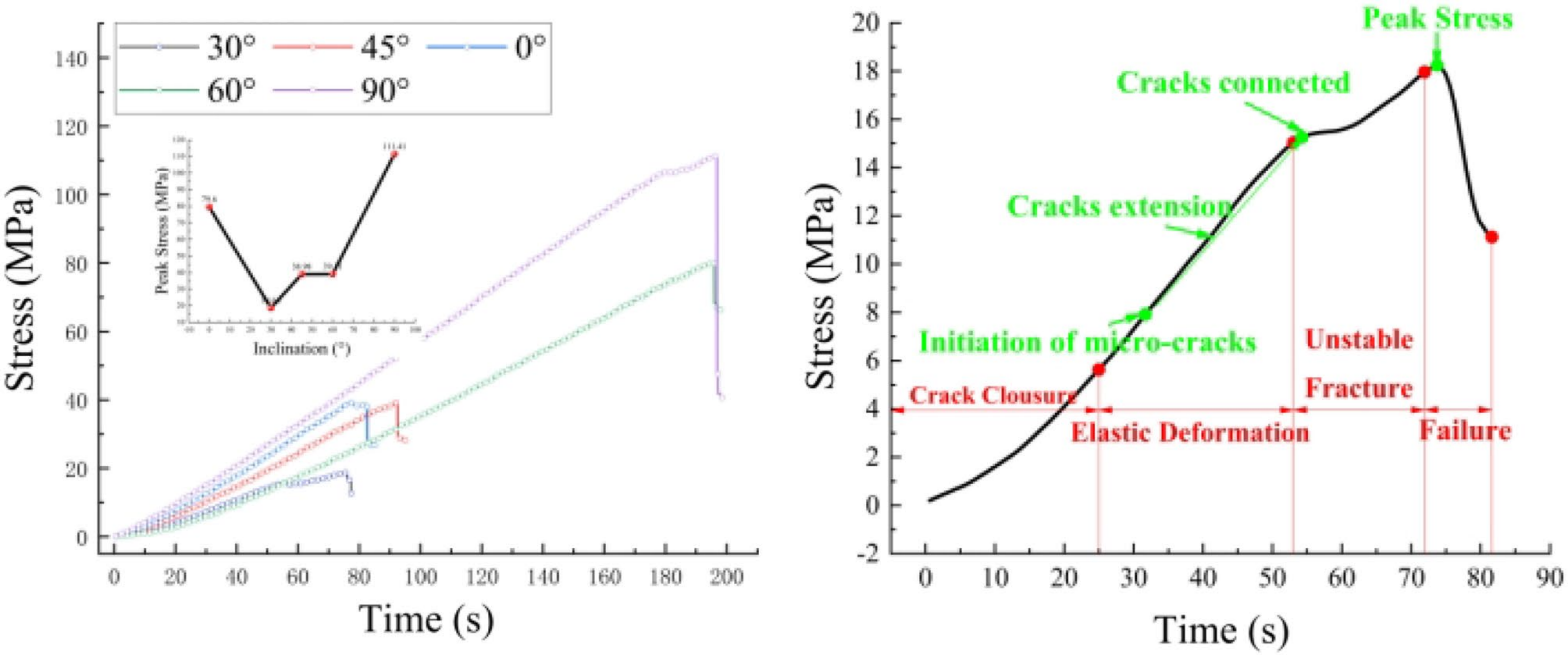
Failure process of phyllite at different angles.

The stress–strain curve of rock is significant for analyzing the changes in rock properties. Because the experiment controls the compression process with a constant displacement rate, the stress-time curve of rock can be used instead of the stress–strain curve. According to each specimen's uniaxial compression failure process (Fig. 4), the rock failure process during the compression process can be divided into four processes^17^: compression stage, elastic stage, instability stage, and failure stage.

- *Stage I* (compression stage) In the early stage of compression, because there are some pores and tiny cracks in the rock itself, in this stage, the stress propagation being blocked, resulting in the stress increases slowly, and the curve shows a trend of upward concave.
- *Stage II* (elastic phase) After entering the elastic phase, the stress shows an almost linear growth relationship because the internal gap has been compacted. Some micro-cracks appear inside the rock, expanding over a range as stress continues to load.
- *Stage III* (instability stage) With the continuous expansion of cracks and the generation of new cracks, some cracks begin to coalesce. The stress-time curve presents nonlinear characteristics, indicating that the destruction of the rock mass is coming.
- *Stage IV* (failure stage) At this stage, a coalescence occurs between the cracks. After the stress peak, penetrating crack appears in the rock mass, and the rock mass is completely broken; the stress occurs cliff-like reduction.

Observe the stress-time curve of different inclination samples, we can find that for the samples with inclination angles of 0° and 90°, the compression process is less affected by the inclination, and the curve has a higher angle; for samples with inclination angles of 30°, 45° and 60°, the joint inclination has stronger control over compressive strength, and the curve has a lower angle. The phenomenon is similar to the previous Wu experiment result^10^.

### Realization of the spatial and temporal distribution of IRT fields and their quantitative analysis

#### Distribution characteristics of the IRT fields in time

**Figure 5:**
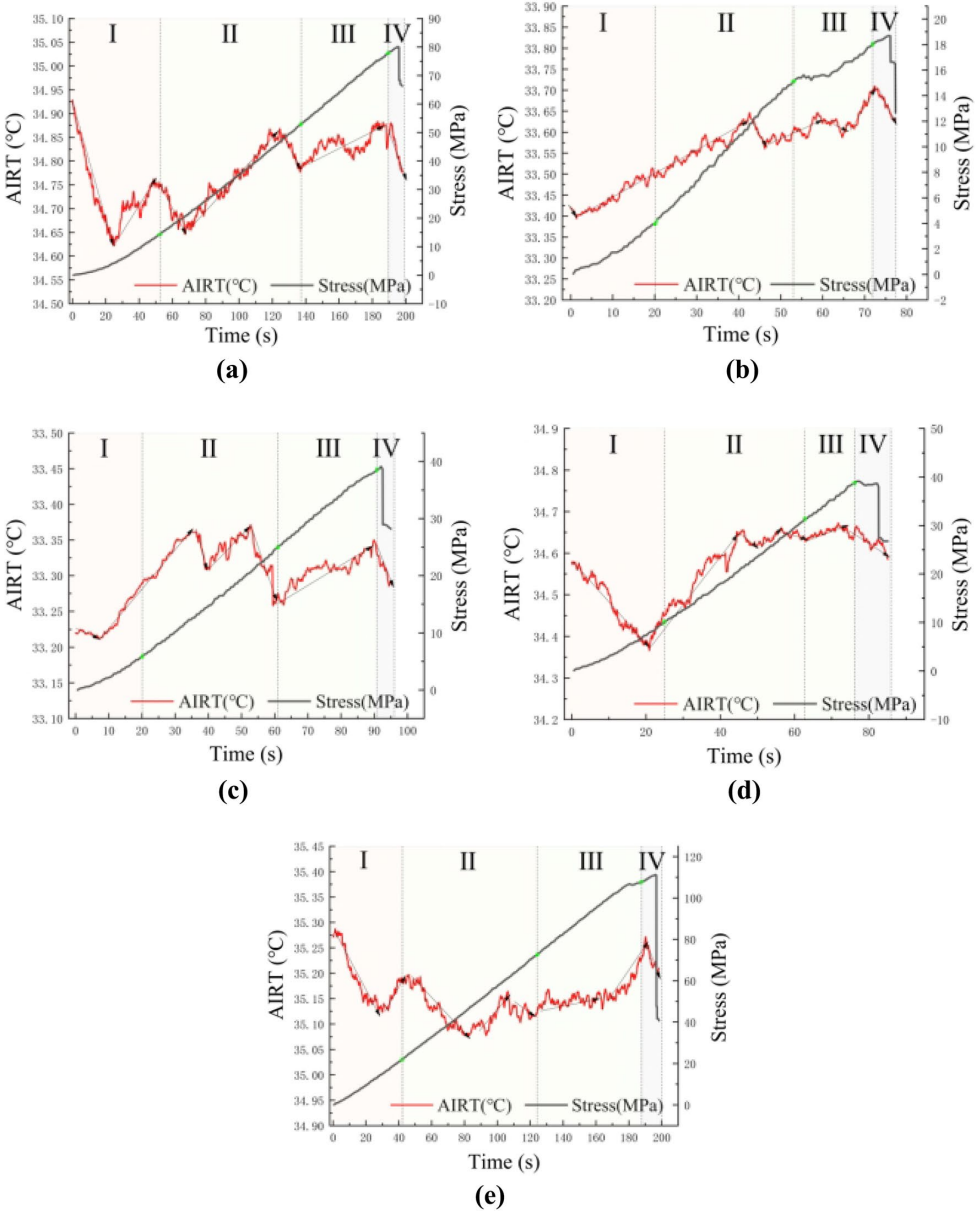
Time distribution of AIRT images: (**a**–**e**) present phyllite specimens with 0°, 30°, 45°, 60°, and 90°, respectively.

By analyzing and comparing the stress-time curve of AIRT images and different samples (Fig. 5), we found that the changing trend of AIRT has a certain correlation for different joint inclination samples, and the AIRT trends of different samples at different stages are also consistent, which is consistent with previous research results^10^. In the process of sample destruction, according to the changing trend of AIRT, combined with the thermal frictional effect, disruptive effect, and thermoelasticity, the change process of AIRT under the four stages is explained:

- *Stage I* (compression stage) In this stage, because the rock mass has some pores and cracks, in the process of external pressure, the internal gas is squeezed out of the rock mass. While the gas escapes from the rock mass, it also takes away some heat, causing the AIRT to show a downward temperature trend. As the increasing load continues, the internal gas is almost eliminated, the overall temperature rises, and the AIRT shows the trend of gradually increasing.
- *Stage II* (elastic stage) With the increase of load, some micro-cracks appear in the rock mass. However, in the elastic stage, the axial load of the rock is at a low level, and the thermoelastic effect is still dominant in the temperature control, so AIRT shows an upward trend. Different forms of damage mainly cause the fluctuation phenomenon in the upward stage. When the crack type is a shear crack, the temperature will rise due to friction, but the tensile crack will lead to a decrease of AIRT.
- *Stage III* (plastic stage) As the load continues to increase, the crack expansion, coalescent, control ability of structural weak plane to rock is stronger and stronger, the formation of cracks is mainly shear cracks, the frictional effect between the crack surface is also increasing with the increase of load. The overall macroscopic performance of the rocks was an elevated AIRT.
- *Stage IV* (failure stage) With the clear fracture sound and the splash of rock debris, the penetrating cracks in the rock mass make it difficult to transmit the axial stress, and the frictional effect between the cracks is correspondingly weakened. The macroscopic performance is the AIRT decline.

#### Characteristics of the distribution of the IRT fields in space

**Figure 6:**
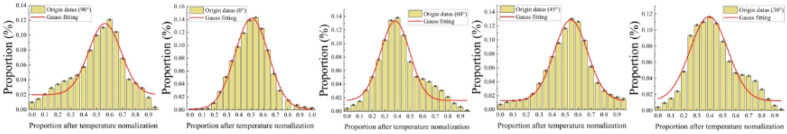
Spatial distribution diagram of the IRT fields.

Each element on the spatial matrix A is counted and the statistical results are made into histograms to obtain the spatial distribution of the thermal image of each sample (Fig. 6). According to the distribution and fitting of IRT shown in the figure, the spatial distribution of IRT conforms to Gaussian distribution, and its expression is shown as follows:

6 $$ y = y_{0} + \frac{A}{{w\sqrt {\pi /2} }}e^{{ - 2\frac{{\left( {x - x_{c} } \right)^{2} }}{{w^{2} }}}} $$

For the fitting results of IRT distribution of samples with different inclination angles, the parameters are shown in the Table 3. In addition, the Gaussian curve and the curve fitting degree is very high, the curve variance range (0.9281–0.9904). But for different samples, the IRT distribution is different. For samples with joint inclination of 30°, the spatial distribution is symmetrical. For the samples with joint dip angles of 90° and 45°, the peak value is slightly shifted to the right, while the samples with joint dip angles of 60° and 0° are slightly shifted to the left. When the joint dip angle is 0–60°, with the increase of bedding inclination angle, the proportion of temperature away from the symmetry center gradually decreases, and the temperature distribution is more concentrated, This is because, with the increase of joint inclination angle, the proportion of rock mass cracks along the joint surface damage to the total cracks increases, which increases the difference between the friction effect of the joint surface and the radiation intensity of other parts, resulting in the concentration of the overall distribution; similarly, when the joint inclination angle exceeds 60°, the temperature distribution gradually moves away from the symmetry center.

**Table 3:** Fitting results and its variance.

Layer inclination (°)	0	30	45	60	90
y0	0.17097	1.72506	2.13005	2.7279	3.56443
xc	0.5433	0.4136	0.57337	0.39639	0.59558
w	0.29935	0.32112	0.26848	0.21274	0.23911
A	9.36591	8.35887	6.81939	5.85173	5.77456
Variance	0.9904	0.93156	0.95427	0.94602	0.9281

### Discrete characteristics of IRT field at different stages

**Figure 7:**
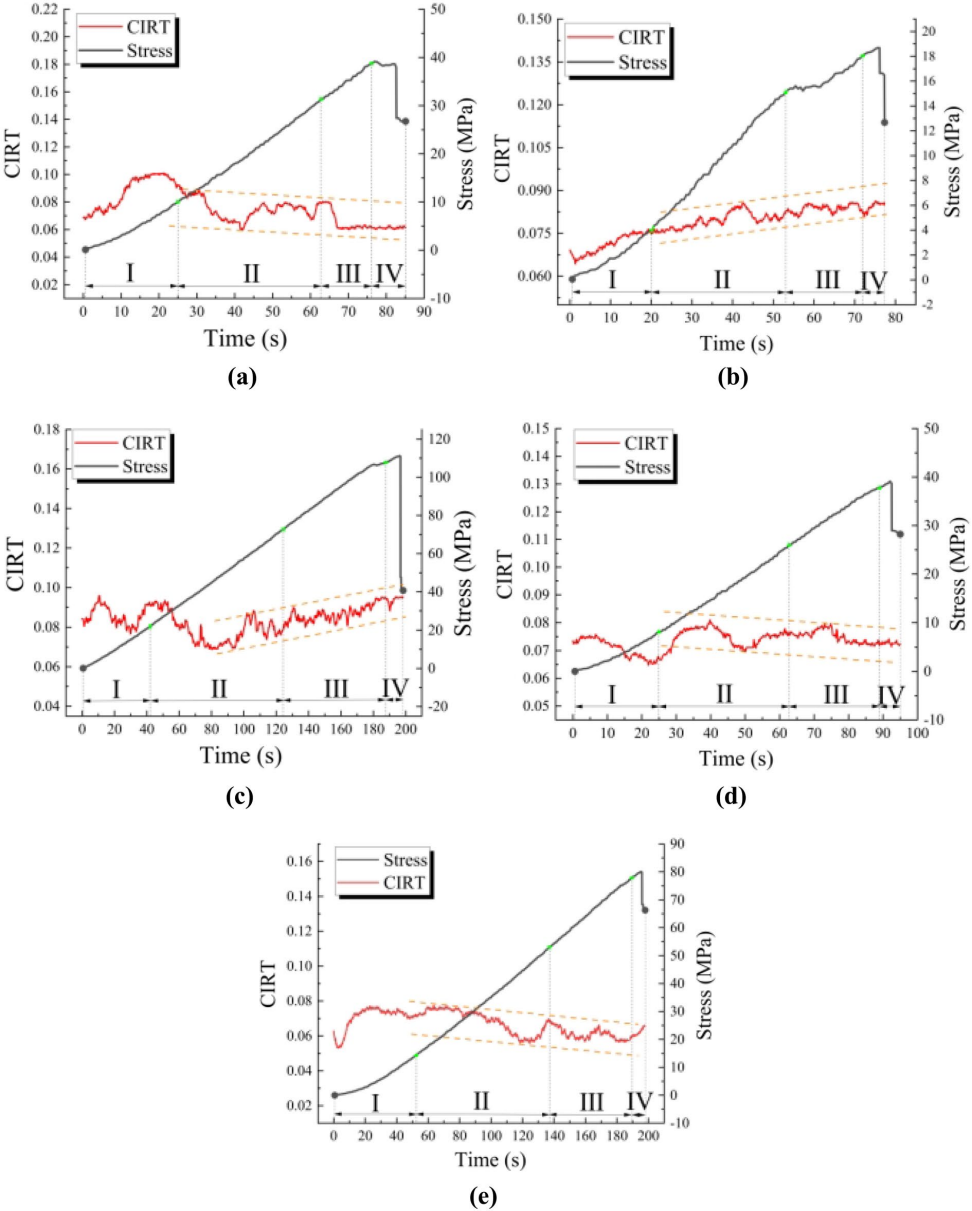
Time distribution of CIRT images: (**a**–**e**) present phyllite specimens with 0°, 30°, 45°, 60°, and 90°, respectively.

According to the temperature information of each part of different specimens, we use CIRT quantitative analysis index to capture the evolution process of sample dispersion in time. According to the statistical results (Fig. 7), CIRT curve shows a certain evolution law in time. As follows:

- *Stage I* (compression stage) the CIRT curves of different samples all showed a ‘U’ type change process. The main reason is that in the compression stage, the small defects on the surface are gradually compacted, and the difference between the surface emissivity of different parts of the specimen is decreasing. According to the Stefan-Boltzmann formula, the thermal radiation ability of the sample surface is similar, the difference of the temperature information captured is small, and the CIRT curve of the macroscopic performance decreases. After the compaction of small cracks on the surface, due to the thermoelastic effect, a discrete small-scale high-temperature region appears in some areas of the sample, and the CIRT curve decreases macroscopically.
- *Stage II* (elastic stage), with the continuous loading of the sample, the thermal radiation of the rock mass is mainly controlled by the thermoelastic effect. At this stage, the changing trend of different samples is different, but the CIRT fluctuates within a specific range.
- *Stage III* (instability stage), the CIRT fluctuation amplitude of the sample gradually decreases, and the curve area steadily rises or decreases, which corresponds to the crack expansion part in the failure process.
- *Stage IV* (failure stage), as the rock mass is destroyed, the friction effect is weakened, the CIRT curve changes smoothly, reflecting that the overall temperature distribution of the rock is relatively uniform.

### Prediction of rock mass failure time

#### Prediction of rock mass destruction time by using neural networks

According to the results of “Establishment of the IRT field quantitative indicators” section, different quantitative analysis indexes have a specific time change in the process of rock failure. For example, before rock mass failure, AIRT increases sharply due to the violent acts of internal shear cracks, reaching a peak before the rock mass is wholly destroyed; before entering the plastic stage, the expansion of tensile cracks leads to the overall downward trend of AIRT. Therefore, by extracting the information of different quantitative analysis indexes (CIRT, $\Delta$, AIRT) in time and space ($\Delta$ is the difference between the maximum temperature and the minimum temperature in the thermal image, shown as formula 7) to predict the stress curve of rock, the prediction of rock failure time can be realized to a certain extent.

7 $$ \Delta = \frac{{\left( {Tt - Tp} \right)}}{Tt} \times 100\% $$

#### LSTM recurrent neural network with attention mechanism

As we all know, LSTM is widely used to analyze non-stationary data sets. Compared with RNN, its main feature is to set up a memory unit, which mainly includes three parts: (1) forgetting gate: which determines the information that needs to be forgotten or lost; (2) Memory gate: which determines what kind of new information needs to be retained in the unit module; (3) Output gate: determining which parts of the cell state need to be output. The memory unit calculation formula of LSTM is as follows :

8 $${\mathrm{f}}_{\mathrm{t}}=\upsigma ({\mathrm{W}}_{\mathrm{f}}\cdot [{\mathrm{h}}_{\mathrm{t}-1},{\mathrm{x}}_{\mathrm{t}}]+{\mathrm{b}}_{\mathrm{f}})$$

9 $${\mathrm{i}}_{\mathrm{t}}=\upsigma ({\mathrm{W}}_{\mathrm{i}}\cdot [{\mathrm{h}}_{\mathrm{t}-1},{\mathrm{x}}_{\mathrm{t}}]+{\mathrm{b}}_{\mathrm{i}})$$

10 $${\mathrm{o}}_{\mathrm{o}}=\upsigma ({\mathrm{W}}_{\mathrm{o}}\cdot [{\mathrm{h}}_{\mathrm{t}-1},{\mathrm{x}}_{\mathrm{t}}]+{\mathrm{b}}_{\mathrm{o}})$$

11 $$ {\text{c}}_{{\text{t}}} = {\text{f}}_{{\text{t}}} \odot {\text{c}}_{{{\text{t}} - 1}} + {\text{i}}_{{\text{t}}} \odot \tanh \left( {{\text{w}}_{{\text{c}}} \left[ {{\text{h}}_{{{\text{t}} - 1}} ,{\text{x}}_{{\text{t}}} } \right] + {\text{b}}_{{\text{c}}} } \right) $$

12 $$ {\text{h}}_{{\text{t}}} = {\text{o}}_{{\text{t}}} \odot \tanh \left( {{\text{c}}_{{\text{t}}} } \right) $$

where represents the output of the previous module unit; the input vector at time t; represents the bias matrix; represents the sigmoid activation function.

After adding the Attention mechanism, the model can assign different weights to different thermal imaging analysis indicators, strengthen the long-distance information in the data, retain important information, and improve the model's accuracy. In our model, we transfer the output of the LSTM network to the Attention layer, calculate the scores of the three analysis indicators, give different weights, and finally transfer to the fully connected layer for calculation.

#### Model construction

**Figure 8:**
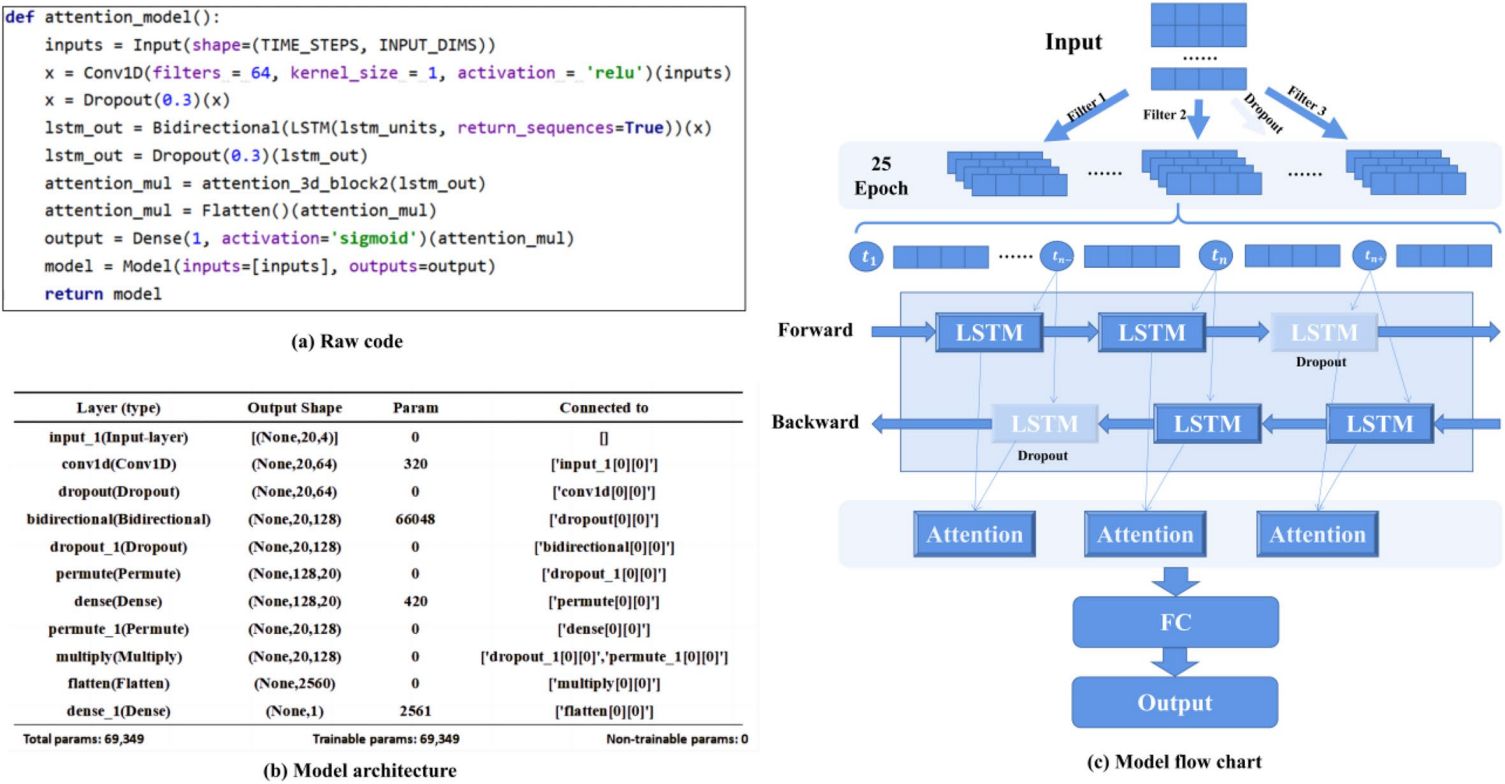
Model framework and its flow chart.

For non-stationary data, compared with traditional time series analysis methods (ARIMA, VAR, etc.), deep neural networks can analyze and predict it well. This paper combines CNN with Bi-LSTM neural network and integrates the Attention mechanism to weigh the output of Bi-LSTM and capture the critical information in the data. We use Tensorflow to build our model and use NVIDIA A16 to train the model. The initial learning rate and Batch-size are 0.01 and 20, respectively. The model’s input is a vector of 4 lengths, and the corresponding output is the stress of the next time step. We choose ADAM and MSE as the optimizer and loss function, respectively. Each experiment was repeated three times, and the average value was taken as our training result. The specific model structure is shown in Fig. 8.

#### Training results

For the relevant results, this paper uses the mean square error to evaluate the experimental results. The mean square error MSE represents the average sum of squares of the distance from the actual value of each data, reflecting the overall distribution of the data. The expression is as follows:

13 $$\mathrm{MSE}=\frac{\sum_{\mathrm{i}=1}^{\mathrm{N}}{\updelta }^{2}}{\mathrm{N}}$$

In the formula, N represents the number of samples, the ith sample, the average of the total samples, and δ represents the difference between the actual value and the predicted value.)

**Figure 9:**
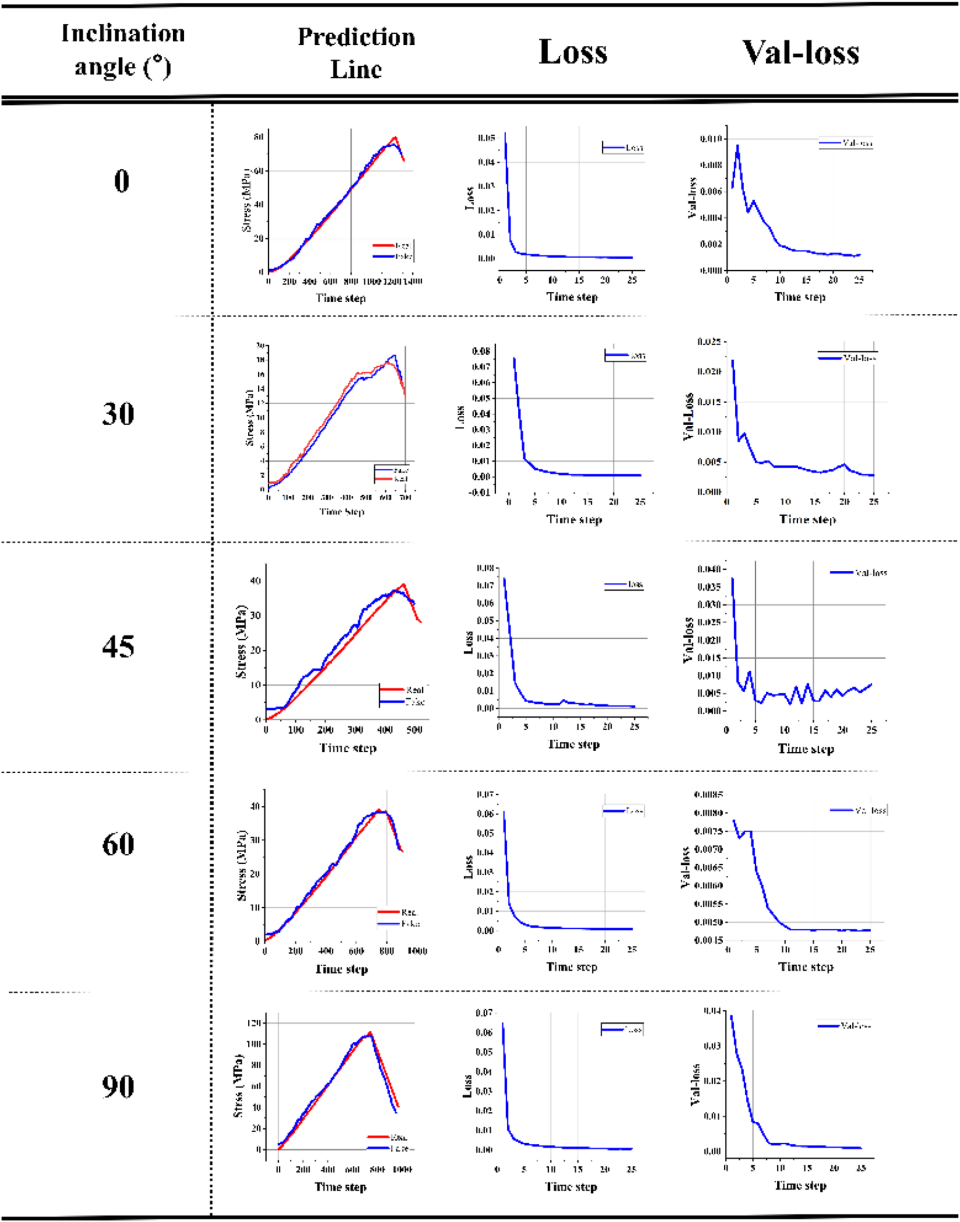
training results.

Figure 9 and Table 4 shows the fitting curve after training and training data, training data error, and verification data error results. The prediction results are consistent with the rock stress curve, and the prediction error at the failure time point is small. The loss of the training set and validation set is at a low level. Therefore, the model can effectively predict the rock failure time.

**Table 4:** Test sample prediction results.

Type of model	Solution of each model				Average of solutions		Best solution		Training time (ms)
	Sample ID	Spend time (ms)	MSE	Relative error (%)	MSE train	MSE val	MSE train	MSE val	
CNN + LSTM	1	288	3.102e−4	3.48	2.887e−4	5.589e−4	9.845e−5	1.956e−4	174
	2	275	2.311e−4	5.16	2.184e−4	5.203e−4	1.235e−4	1.537–4	255
	3	301	1.364e−4	4.96	1.861e−4	3.146e−4	1.128e−4	8.963–5	225
	4	296	5.315e−4	6.78	3.562e−4	8.764e−4	4.123e−5	9.878e−5	194
	5	277	4.562e−4	6.53	3.651e−4	6.512e−4	8.395e−5	8.963e−5	188
CNN + BiLSTM + Attention	1	223	1.031e−5	2.63	2.110e−5	3.132e−5	1.156e−5	2.444e−5	104
	2	210	3.796e−5	4.35	2.815e−5	4.896e−5	2.659e−5	3.575e−5	145
	3	237	4.196e−5	4.82	1.563e−5	6.889e−5	1.196e−5	1.830e−5	137
	4	188	2.237e−5	2.59	2.351e−5	9.852e−5	1.630e−5	1.895e−5	123
	5	215	2.182e−5	1.55	1.565e−5	4.983e−5	1.322e−5	1.177e−5	132

## Discussion

### Rock damage detection by using thermal imaging technology

In terms of crack detection, for the current primary acoustic emission (AE) and microseismic (MS) monitoring, thermal imaging technology, as a non-destructive detection method, provides a brand new direction for rock crack detection by recording the blackbody radiation emitted from the rock mass surface. Although there is no clear conclusion between rock cracking behavior and thermal radiation, many current studies have shown the correlation between the two and proposed various schemes to improve detection accuracy.

### Supplement of the thermal radiation phenomenon during rock damage

The dominant view of rock temperature change is the gas desorption-escape effect, thermoelastic effect, and frictional effect. For rocks with different joint inclination, the gas desorption-escape effect mainly exists in the early stage of the applied load, when there are many voids in the rock. During the compression process, the gas gradually escapes from the rock and takes away the heat; The thermoelastic effect dominates the late phase of the compression stage and the anterior phase of the elastic stage. Due to the low external load, the friction and dissipation between rock cracks have less energy; In the late elastic stage and the plastic stage, Due to the expanding coalescence of the internal cracks, thermal energy resulting from friction collisions between cracks dominates the temperature variation. The cracks mainly have shear cracks and tensile cracks. It is worth noting that the thermal effect caused by the rock joint inclination angle is also different. For the rock with a too small or too large inclination, the main failure mode is the tension failure, and the crack is the tensile crack; during the expansion, the surfaces of the two cracks are separated along the direction of the normal phase, resulting in volume expansion and a weak friction effect. When the joint inclination is (30–60)°, the failure mode is mainly shear failure, and the mode of crack is shear crack. The crack surface slips along the weak surface of the structure, and the friction effect is strong during expansion, resulting in an increase in temperature^7,36^.

To improve the environment’s identification, we slightly moistened the specimen surface in this experiment. At the end of the compaction and early elastic stage, water evaporation takes away part of the heat on the surface. The macro performance of AIRT is declining, but after a short time, with the complete gasification of water, the influence of water is gradually decreased in AIRT. Compared with the previous research results^20,30,33,37^, the previous wetting treatment did not affect the subsequent AIRT curve trend after water evaporation.

### Selection of IRT field quantitative analysis indicators

For the quantitative analysis index of the IRT field, MAXIRT and MINIRT index, in describing the temperature characteristics of rock destruction behavior, due to the environmental disturbance, can not effectively and comprehensively reflect the actual process.However, only the three stages before rock failure, loading process, and after failure can be distinguished; that is, the change in the loading process cannot be effectively analyzed.

In the use of CIRT analysis^21^, for the rock with a certain joint inclination angle, the difference between the rock with prefabricated cracks is that there will not be a strong stress concentration phenomenon during compression, resulting in a strong thermal effect on the rock. The dispersion and skewness of the temperature distribution will not change abruptly, so it cannot effectively predict the time of rock destruction. Moreover, AIRT can reflect the distribution characteristics of the whole IRT field, and has apparent changes in different stages of damage, can effectively analyze the rock damage process in a given area, but using AIRT points, need a certain amount of temperature data to support, for the damage analysis of small areas, using the method of AIRT analysis is not applicable.

In summary, the selection of a single quantitative analysis index cannot be applied to all cases. Only by combining multiple analysis indicators can we better analyze the characteristics of rock failure, which is also the main reason for our subsequent multi-factor joint prediction.

### Model comparison

**Figure 10:**
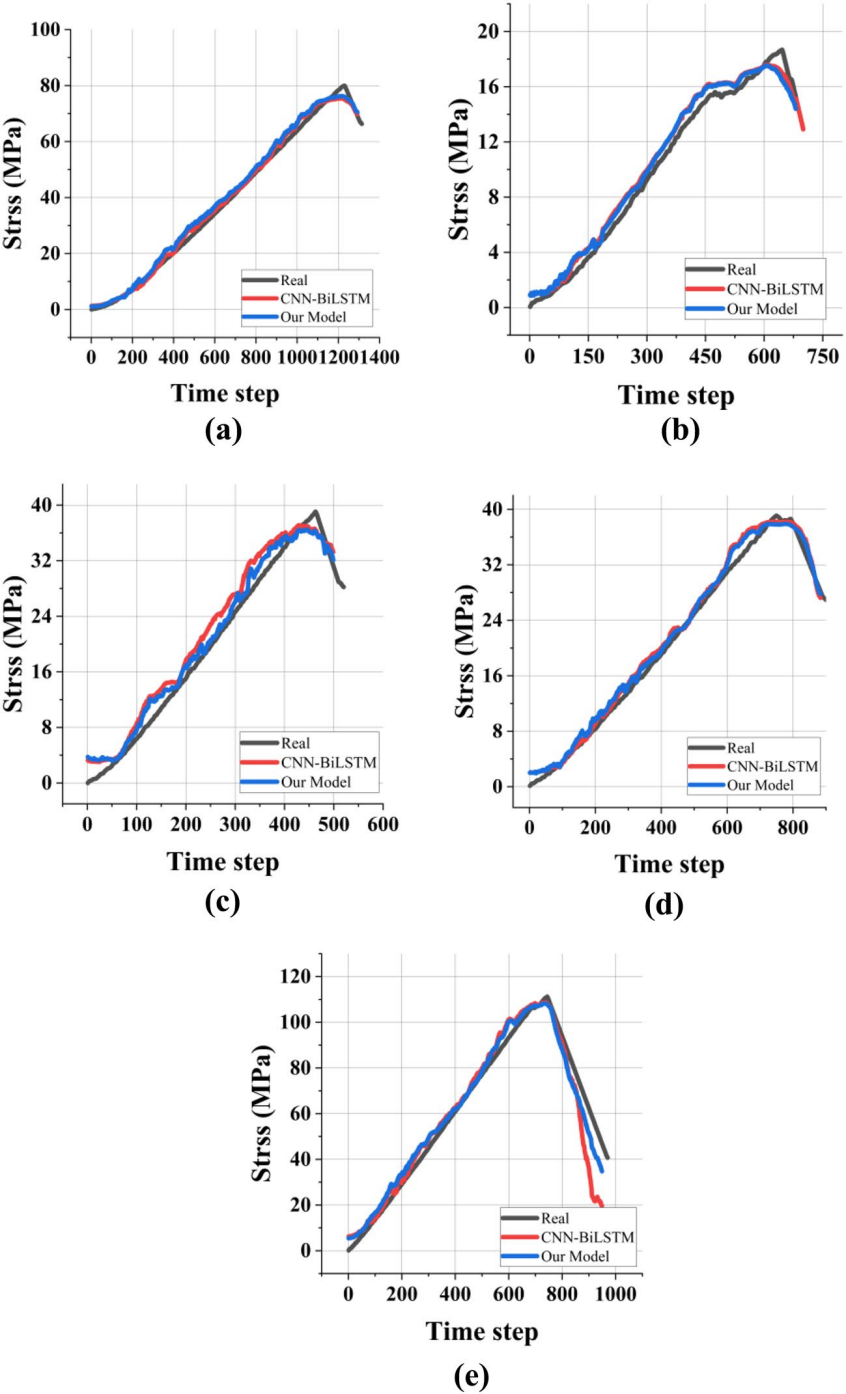
Model comparison results: (**a**–**e**) present phyllite specimens with 0°, 30°, 45°, 60°, and 90°, respectively.

In order to better demonstrate the advantages of our model, we designed a Bi-LSTM model without the Attention mechanism and compared it with our model. The results are shown in Fig. 10 and Table 4. The model results show that our model has better prediction results in most samples than the CNN + BiLSTM network architecture without the Attention mechanism, variance, and relative error. Therefore, our model can predict the rock failure time better than the ordinary time series prediction network.

### The significance of predicting the time of rock destruction

The destruction process of the rock is the process of generation, expansion, coalescence, and final penetration of the micro-cracks inside the rock. By studying the failure process of rock, the corresponding protective measures are put forward to inhibit the evolution process of micro-cracks, prevent the occurrence of rock failure, and reduce the loss caused by rock failure, which is the center of the study. Predicting the occurrence of rock damage can better analyze the current state of rock, facilitate the optimal decision, and make corresponding measures according to the situation to achieve the purpose of optimal protection.

## Conclusion

This paper designed a quantitative analysis of the IRT field by Python, combined with Infrared thermal imaging technology and artificial neural network technology. It analyzed and predicted the destruction process of phyllite specimens with different inclination angles. It is found that the stress-time curve of different joint inclination shows a similar evolutionary stage, and the size of the inclination angles also controls the crack type produced by the damage. It provides a theoretical basis for later research. Accordingly, we draw the following conclusions:

- According to the stress change process of the rock, we roughly divided the rock destruction process into four stages: compression, elasticity, plasticity, and failure. In the elastic stage, the dominant factor of rock temperature control gradually changes from the thermoelastic effect to the frictional effect due to the formation and expansion of shear cracks. When the rock enters the failure stage, the friction between the rock will reach the strongest, producing a large amount of heat, which can be used as a sign before the rock is damaged.
- The AIRT index can appropriately describe and analyze the process of the evolution of rock destruction by recording the infrared radiation on the surface of the rock mass and reflecting the internal activity of the rock mass to some extent. For the AIRT distribution of rocks with different inclination angles, the results show that the distribution characteristics of AIRT accord with the Gaussian distribution, and the concentration ratio of the distribution is different, showing the phenomenon of peak deviation.
- The CNN + BiLSM recurrent neural network with Attention mechanism is suitable for predicting rock failure time. Satisfactory results can be obtained for rocks with different dip angles. It can be used as a method to predict rock failure time, which helps accurately judge the rock stage.

## Data Availability

The datasets used and/or analysed during the current study available from the corresponding author on reasonable request.
